# Abnormal perilesional BOLD signal is not correlated with stroke patients’ behavior

**DOI:** 10.3389/fnhum.2013.00669

**Published:** 2013-10-16

**Authors:** Bianca de Haan, Chris Rorden, Hans-Otto Karnath

**Affiliations:** ^1^Center of Neurology, Division of Neuropsychology, Hertie-Institute of Clinical Brain Research, University of TübingenTübingen, Germany; ^2^Department of Psychology, University of South CarolinaColumbia, SC, USA

**Keywords:** perilesional fMRI, spatial neglect, attention, aphasia, neurovascular coupling, interhemispheric imbalance, stroke, human

## Abstract

Several functional magnetic resonance imaging (fMRI) studies of acute stroke have reported that patients with behavioral deficits show abnormal signal in intact regions of the damaged hemisphere close to the lesion border relative to homologous regions of the patient’s intact hemisphere (causing an interhemispheric imbalance) as well as analogous regions in healthy controls. These effects have been interpreted as demonstrating a causal relationship between the abnormal fMRI signal and the pathological behavior. Here we explore an alternative explanation: perhaps the abnormal Blood-Oxygenation Level Dependent (BOLD) fMRI signal is merely a function of distance from the acute lesion. To investigate this hypothesis, we examined three patients with an acute right hemisphere cortical stroke who did not show any overt behavioral deficits, as well as nine healthy elderly controls. We acquired fMRI data while the participants performed a simple visual orientation judgment task. In patients, we observed an abnormal interhemispheric balance consisting of lower levels of percent signal change in perilesional areas of the damaged hemisphere relative to homologous areas in neurologically healthy controls. This suggests that the physiological changes and corresponding interhemispheric imbalance detected by fMRI BOLD in acute stroke observed close to the lesion border may not necessarily reflect changes in the neural function, nor necessarily influence the individuals’ (e.g., attentional) behavior.

## Introduction

Functional magnetic resonance imaging (fMRI) uses a Blood-Oxygenation Level Dependent (BOLD) measure to infer brain activity. BOLD fMRI has provided a robust tool for understanding how the healthy human brain functions. Recently, this technique has also been employed to study individuals who have suffered from brain injury due to stroke. For example, Corbetta et al. (2005) examined 11 stroke patients who had profound spatial neglect in the acute stage of a right hemisphere stroke and observed reduced BOLD signal in intact attention specific regions of the damaged hemisphere relative to homologous regions of the non-damaged hemisphere, with the chronic restoration of this interhemispheric imbalance correlating with improved attentional orienting. Likewise, Saur et al. (2006) examined 14 patients who recovered from acute aphasia after a left hemisphere stroke. Acute and subacute fMRI indicated an initially decreased signal in language specific areas of the damaged hemisphere, followed by increased BOLD signal in both the damaged and the intact hemisphere. Chronic fMRI of these patients showed a reduction of this abnormal BOLD response pattern that was accompanied by language improvement. Both studies thus concordantly reported that acute/subacute stroke was associated with an imbalance of BOLD signal in the structurally intact tissue of the damaged relative to the non damaged hemisphere and that a return to the pattern observed in healthy controls correlated with improved behavior. Importantly, both studies concluded that the patients’ behavioral deficits (i.e., the defects in attentional orienting or in language processing, respectively) did not depend just on the neuronal loss at the site of injury but rather were also causally connected to this abnormal BOLD signal in structurally intact tissue.

However, interpreting such results might be complicated by the fact that BOLD fMRI measures changes in blood flow rather than directly measuring brain activity and that the relationship between brain activity and local hemodynamics (i.e., neurovascular coupling) might be abnormal in stroke patients. This problem is exacerbated by the practice of comparing the BOLD response in stroke patients not to that in control patients with brain injury in the same cerebrovascular territory who do not suffer the behavioral deficit of interest, but to that in neurologically healthy subjects (e.g., Corbetta et al., 2005; Saur et al., 2006) as this practice rests heavily on the assumption that the relationship between neural activity and the BOLD response is comparable in both populations (D’Esposito et al., 2003). As a consequence, it is possible that the observed local differences in BOLD response between stroke patients and healthy controls in these studies cannot be solely attributed to differences in neuronal activity, but might also be due to abnormal neurovascular coupling after stroke, i.e., an abnormal relationship between neuronal activity and hemodynamic regulation in the brain (without changes in neuronal activity), or a combination of these two effects. Should this be the case, then one should expect unusual BOLD fMRI signals in stroke patients independent of the behavioral deficit under study.

Several studies have suggested that neurovascular coupling might be abnormal in stroke patients suffering from an arterial stenosis/occlusion (Carusone et al., 2002; Hamzei et al., 2003; Amemiya et al., 2012). As neurovascular coupling depends amongst other things on cerebral blood flow, this finding is not terribly surprising. However, there are also studies that suggest that neurovascular coupling can be abnormal in stroke patients who show no evidence of an arterial stenosis/occlusion (Krainik et al., 2005). Moreover, seminal research by Rossini et al. (2004) provide compelling evidence that neurovascular coupling may be abnormal in stroke patients despite absence of arterial stenosis/occlusion and presence of neuronal activity. These authors found that in approximately half of the stroke patients they investigated, no BOLD response could be elicited despite the fact that magnetoencephalography (MEG) demonstrated normal neuronal activity in these patients. Additionally, they found that the absence of a BOLD response in these patients was strongly related to impaired cerebrovascular reactivity (i.e., the increase in blood flow in response to a cerebral vasodilator like for example carbon dioxide or acetazolamide).

Interestingly, the interhemispheric imbalance reported by both Corbetta et al. (2005) and Saur et al. (2006) partly occurs in regions close to the lesion border and their homologues. Based on a recent suggestion that cerebrovascular reactivity might be particularly impaired close to the lesion border (Richardson et al., 2011), our objective was to see if the BOLD signal measured in structurally intact tissue of stroke patients might be abnormal simply depending on the distance to the brain lesion. In other words, we aimed to investigate whether the interhemispheric imbalance in areas close to the lesion border (as in the reports by, e.g., Corbetta et al. (2005) and Saur et al. (2006)) might be (partly) due to abnormal neurovascular coupling. Should this be the case, then one would expect abnormal BOLD responses in areas of the brain close to the lesion border also in patients without spatial neglect or aphasia. This would provide an alternative explanation for studies that interpret abnormal BOLD signals at specific locations as causing impaired (cognitive) function after, e.g., a stroke in the territory of the middle cerebral artery. While these are not mutually exclusive effects, evidence of an abnormal BOLD response close to the lesion border in absence of an overt behavioral deficit would emphasize that BOLD effects observed close to the lesion border in acute stroke might be particularly challenging to interpret. Moreover, a demonstration of an association between abnormal BOLD effects and distance from the lesion border would provide important suggestions concerning potential underlying mechanisms.

Specifically, we examined acute neurological right hemisphere stroke patients (similar to Corbetta et al. (2005)) who did not exhibit spatial neglect (whereas the patients studied by Corbetta et al. (2005) did have this disorder). The core question is whether the present patients exhibit abnormal BOLD activity in the damaged hemisphere and/or an abnormal interhemispheric balance, particularly in areas of the brain close to the lesion and their homologues in the intact hemisphere, even though they do not exhibit spatial neglect. In addition, whereas Corbetta et al. (2005) used a spatial attention task, we explicitly chose a visual orientation task where—even in the case of subclinical neglect—our patients should have no specific deficit. Thus, in this visual orientation task, we would expect normal BOLD activity in intact areas with normal neurovascular coupling. Moreover, this visual orientation task has previously been shown to result in widespread bilateral activation in temporo-parieto-frontal areas of the brain (Altmann et al., 2005). These decisions were made to provide a clear test for our hypothesis that the BOLD signal may be disrupted independent of the behavioral impairment or the task used to elicit a response.

## Materials and Methods

### Participants

All subsequently admitted patients suffering an acute cortical right hemispheric stroke were screened at the Tübingen Center of Neurology for potential inclusion in the current study. This resulted in the detection of three patients suitable for inclusion. Each patient had suffered a stroke in the territory of the middle cerebral artery (lesion size 38.48 cubic centimeter (patient 1), 28.46 cubic centimeter (patient 2) and 29.84 cubic centimeter (patient 3)). Inclusion criteria were: right handed, no evidence of older infarcts or white matter disease, no evidence of other neurological or psychiatric disorder, no evidence of hemodynamically relevant (> 50%) stenoses in either Doppler sonogram or angiography, and no evidence of spatial neglect and/or extinction during a clinical assessment performed on the same day as the fMRI study that included the bells cancellation test (center of cancellation (CoC; Rorden and Karnath, 2010) score 0, −0.015 and 0 for patient 1, 2 and 3 respectively), the letter cancellation test (CoC score 0 in all patients), the copying task (score 0 in all patients, scored as described in Ferber and Karnath, 2001), the line bisection task (percent deviation 3.33, 3.58 and −2.75 for patient 1, 2 and 3 respectively, scored as described in Ferber and Karnath, 2001) and a fingerperimetrical assessment of visual extinction (no contralesional omissions during either bilateral or unilateral stimulation in any of the patients). These diagnostic tests for neglect were virtually identical to the tests used by Corbetta et al. (2005), with the exception that we used the bells cancellation test instead of the star cancellation test as the bells cancellation test has been shown to be more sensitive to the presence of neglect (Ferber and Karnath, 2001). Moreover, we used the copying task and the line bisection task instead of the reading test from the Behavioral Inattention Test that Corbetta et al. (2005) used. Furthermore, during their entire stay on the neurological ward, the patients were observed by our team of neurologists, nurses, clinical neuropsychologists and physiotherapists. In addition to our formal neglect screening, none of the involved therapists noted any behavioral evidence of clinical neglect in activities of daily living. The age of the patients who volunteered was 59, 64, and 69 years for patient 1, 2 and 3 respectively (all male) and the time between stroke onset and the fMRI study was 2, 4 and 6 days. Additionally, ten right handed elderly subjects (mean age 61 years old, range 51–70 years, four male) participated in the present study. The elderly subjects had no history of neurological or psychiatrical disorders, and had normal or corrected to normal vision. All participants signed an informed consent, approved by the ethics committee of the Medical Faculty of Tübingen. The neurologically healthy elderly subjects were paid for participation in the study. The study was performed in accordance with the Declaration of Helsinki.

### Task design and procedure

All participants performed a task based on the visual orientation judgment task developed by Altmann et al. (2005) while lying in the scanner. E-prime software (Psychology Software Tools Inc.) was used to present the stimuli and record behavioral responses. Visual stimuli were projected on a screen positioned at the head of the magnet bore which subjects viewed via a mirror mounted on the head coil. The visual stimulus set contained 37 images of both animals and man-made objects, provided courtesy of Michael J. Tarr (Brown University, Providence, RI). All images were presented with a size of 9.5˚ visual angle and were symmetrical around the vertical midline. In each trial, participants were shown a centrally presented upright or inverted image of an object for a duration of 500 ms. After a gap of 1000 ms in which a central fixation cross was presented, participants were again shown an image of the same object as before for a duration of 500 ms, which was either presented in the same orientation as the first image or was rotated by 180˚. Participants were instructed to respond by pressing a button with their right index finger if the orientation of both images was identical (i.e., either both upright or both inverted) within a time limit of 2000 ms. Subsequently, successive trials were separated by a variable inter-trial interval ranging between 2000 and 12,000 ms (mean 3900 ms) following a roughly exponential distribution during which the central fixation cross was again presented. The task was divided in three runs each containing 40 trials and all participants performed three runs. Within each run, each trial type (upright-upright, upright-inverted, inverted-upright and inverted-inverted) appeared equally often and the order in which the trials were presented was randomized. Prior to performing the task in the scanner, participants performed a practice run. All participants (both patients and elderly neurologically healthy subjects) included in the study demonstrated that they understood and were able to adequately perform the task during this practice run.

### Imaging and data analysis

All functional imaging was acquired using a three Tesla Siemens Magnetom Trio scanner (Erlangen, Germany). Three sessions of continuous fMRI data were collected (one for each task run) for each neurologically healthy subject and each neurological patient. Each session consisted of 118 whole brain functional T2* echo-planar imaging (EPI) volumes collected axially with a flip angle of 90˚, a time to echo (TE) of 40 ms, a time to repetition (TR) of 2.69 s. Each fMRI volume included 33 slices acquired in sequential ascending order with a slice thickness of 3 mm (with no gap between slices) and an in-plane resolution of 3 × 3 mm (field of view (FOV) = 192 × 192). We also acquired T1-weighted structural scans from all participants except one of the patients (for whom we were unable to collect a T1-weighted volume due to time constraints) to aid normalization (176 slices, 1 × 1 × 1 mm). In addition, T2 fluid attenuated inversion recovery (T2-FLAIR) sequences were acquired from all patients to map lesion location.

Preprocessing and statistical analyses were performed in SPM8.^1^ In the neurological patients, the boundary of the lesion was delineated directly on the individual T2-FLAIR image for every single transverse slice using MRIcroN software.^2^ For the patients (except the patient without a T1-weighted volume) both the T2-FLAIR volume and the lesion shape were coregistered with the T1-weighted volume (Collignon et al., 1995). In both the neurologically healthy subjects and the stroke patients, the functional volumes were slice time corrected using the middle slice as the reference slice (Henson et al., 1999) and realigned to match the first volume of the first session (Friston et al., 1995). Due to scan-to-scan movement that exceeded 3mm, one neurologically healthy subject (male, 51 years old) was excluded from further analysis, thus leaving nine neurologically healthy subjects for further analysis. Subsequently, the T1-weighted volume and, for the patients, the T2-FLAIR volume and the lesion shape were coregistered with the mean functional volume obtained after realignment (Collignon et al., 1995). For both the neurologically healthy subjects and the patients (except the patient without a T1-weighted volume), transforms for warping the coregistered T1-weighted volume to standard stereotaxic space were computed by unified normalization-segmentation (Ashburner and Friston, 2005), using age-appropriate priors obtained from the “Clinical Toolbox” for SPM8 (Rorden et al., 2012). The resulting transformation parameters were used to warp the functional volumes, structural volumes and—for the patients—the lesion map into stereotaxic space. For the patient without a T1-weighted volume, the T2-FLAIR volume and lesion map were mapped into stereotaxic space by matching the T2-FLAIR to the gg-flair-181-asym T2-FLAIR template provided by the Glahn group^3^ using the normalization algorithm provided by SPM8 (Ashburner and Friston, 1999) and the resulting transformation parameters were used to warp the functional volumes into stereotaxic space. In all patients, cost–function masking was employed for determination of the transformation parameters (Brett et al., 2001). Finally, the functional volumes of both neurologically healthy subjects and neurological patients were smoothed with an isotropic 8 mm full width half maximum (FWHM) Gaussian filter (Worsley and Friston, 1995). Task-related changes in blood-oxygenation level were estimated for a period of nine TRs (24.21 s) with a Finite Impulse Response (FIR) function, which does not assume a canonical shape for the hemodynamic response function (Miezin et al., 2000). Each FIR period was equivalent to 1 TR or 2.69 s.

**Figure 1:**
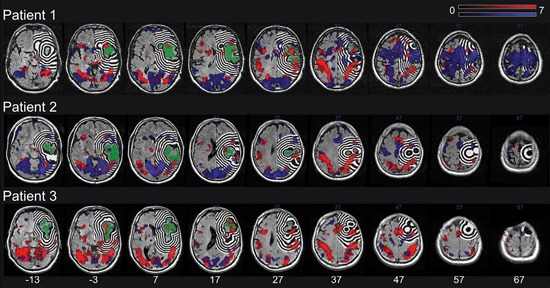
**The lesion (green) and the dilation of this lesion into twelve adjacent 3 mm right hemispheric perilesional regions for each patient.** Additionally shown are the results of the statistical analysis highlighting the voxels showing significant task related changes (regardless of trial type) in the individual patient (blue) as well as the group of three control subjects assigned to the respective patient (red). The lesion shape, perilesional regions and the results of the statistical analyses are plotted onto the patients’ T2-FLAIR image. All images are in neurological orientation and a significance threshold of 0.05 FDR corrected for multiple comparisons was used.

Utilizing a custom feature added to the MRIcroN software, each patient’s normalized lesion shape was dilated into twelve adjacent 3 mm perilesional regions expanding 39 mm beyond the lesion’s rim (i.e., 3–6 mm, 6–9 mm, 9–12 mm etc.; see Figure 1). To account for partial lesion volume, the first perilesional region started 3 mm from the lesion border. Further, the twelve dilated regions were binary (i.e., a voxel was either part of the region or not) and successive regions identified mutually exclusive voxels (e.g., the first region included voxels further than 3 mm upto 6 mm, the second region was further than 6 mm up to 9 mm, etc). In addition, the perilesional regions were left-right flipped to create the homologuous perilesional regions for the intact left hemisphere. The twelve perilesional regions in both the left and the right hemisphere were first multiplied with a mask including all left or right hemispheric voxels and subsequently masked with the results of a statistical analysis highlighting the voxels showing significant task related changes (regardless of trial type) in a subset of the group of neurologically healthy subjects. Specifically, we randomly assigned three of the neurologically healthy subjects to each patient to serve as control subjects for that patient with the remaining six neurologically healthy subjects used to determine the voxels that show significant task related changes. This was done to avoid bias by ensuring that the data used to select the voxels showing task related changes was independent from the data used in the later comparisons between patients and control subjects. These voxels showing significant task related changes were assessed by a voxelwise repeated-measures analysis of variance (ANOVA) with FIR time bin (1–9 TRs) as within subject factor using the smoothed functional volumes as input. Voxels affected by the task were identified by assessing the main effect of FIR time bin, i.e., the voxels where the BOLD signal in the FIR time bins differed significantly. The resulting F statistics were corrected for multiple comparisons using a voxelwise False Discovery Rate (FDR) correction (Benjamini and Hochberg, 1995; Genovese et al., 2002). Voxels were considered significant at *q* < 0.05. Masking the perilesional regions of the left and the right hemisphere with the results of the statistical analysis highlighting voxels that showed significant task related changes thus resulted in twelve perilesional regions reflecting task responsive voxels for both the intact left and the damaged right hemisphere. For patient 1, the volume of the perilesional regions closest to the lesion and their homologues in the intact hemisphere tended to be larger than the volume of the perilesional regions most distant to the lesions and their homologues in the intact hemisphere. For patients two and three the volume of the perilesional regions closest to the lesion and their homologues in the intact hemisphere was approximately 2–3 times smaller than the volume of the perilesional regions most distant to the lesion and their homologues in the intact hemisphere. Importantly, however, the volume of the smallest perilesional regions was still 1237 mm³ ensuring adequate signal to noise in all perilesional regions. Finally, for each patient the SPM Toolbox “MarsBaR”^4^ was used to calculate the percentage signal change in each of the resulting perilesional regions reflecting task responsive voxels for both the intact left and the damaged right hemisphere. To avoid smoothing artifacts in perilesional regions close to the lesion border in the patients, the calculation of the percentage signal change was based on the unsmoothed fMRI data. For each patient, this percentage signal change was compared to the percentage signal change in the same voxels in the three control subjects allocated to that patient.

## Results

### Behavioral results

Two-sample *t*-tests demonstrated no significant differences between patients and neurologically healthy subjects in reaction times (618 ms (SD 40 ms) for the patients vs. 677 ms (SD 126 ms) for the controls; *t*_10_ = 1.23, *p* = 0.248) or response accuracies (72% (SD 7%) for the patients vs. 83% (SD 17%) for the controls; *t*_10_ = 1.60, *p* = 0.146).

### fMRI results

The result of the statistical analysis highlighting the voxels showing significant task related changes (regardless of trial type) in each individual patient as well as in the subset of the group of neurologically healthy subjects assigned to the respective patient is shown in Figure 1. In each of the three subsets of the group of neurologically healthy subjects, visual orientation judgment was associated with a widespread pattern of activation in both dorsal fronto-parietal cortical areas and more ventrally located occipito-temporal cortical areas, broadly in line with the pattern reported by Altmann et al. (2005).

**Figure 2:**
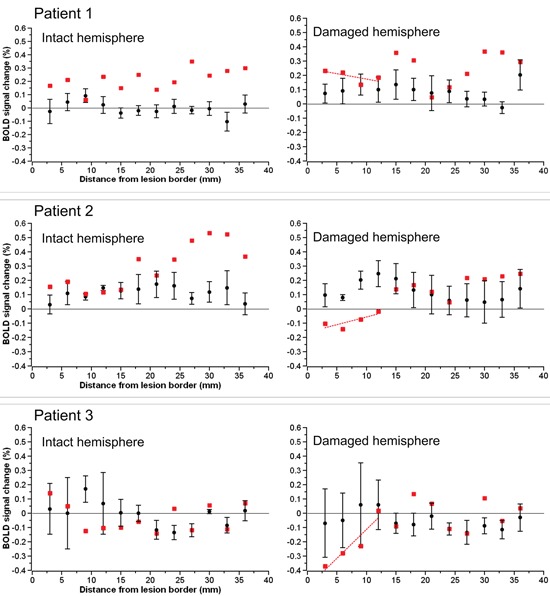
**The BOLD percent signal change in each 3 mm perilesional region for each of the three stroke patients (red squares) as well as the group of control subjects (black circles).** The images on the left depict the data for the left hemispheric perilesional regions and the images on the right the data for the right hemispheric perilesional regions. Error bars reflect standard error of the mean.

**Figure 3:**
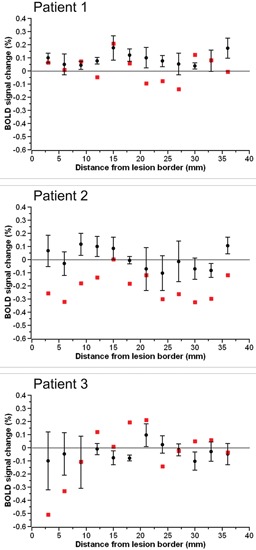
**The interhemispheric imbalance score in each 3 mm perilesional region for each of the three stroke patients (red squares) as well as the group of three control subjects assigned to the respective patient (black circles).** Error bars reflect standard error of the mean.

The BOLD percent signal change in each 3 mm perilesional region for the three stroke patients as well as the group of three control subjects assigned to each patient is shown in Figure 2. In two of the three stroke patients, the percent signal change was noticeably reduced in areas of the brain near the lesion border when compared to the percent signal change in the same regions in controls. Additionally, the subtraction of the percent signal change in the left hemispheric perilesional homologue regions from the percent signal change in the right hemispheric perilesional regions, illustrating the interhemispheric imbalance, is shown in Figure 3. Positive interhemispheric imbalance scores thus reflect a higher percent signal change in the right hemisphere than in the left hemisphere. It can be seen that whereas the controls on average showed a somewhat higher percent signal change in the right than in the left hemisphere, two of the three cortical patients showed the reverse pattern, namely a lower percent signal change in the right (damaged) than in the left (non damaged) hemisphere, particularly in areas of the brain near the lesion border and their homologues.

As the interpretation of statistical analyses performed on our small sample is difficult, we first analyzed the data descriptively. For this descriptive analysis, we collated the signal over each four adjacent perilesional regions creating three—namely near, middle and far—perilesional “super-regions” for each patient in each hemisphere. Subsequently, we calculated for each perilesional “super-region” the mean percent signal change and its range for both the patients and the control subjects for both the left and the right hemisphere. Moreover, for both patients and control subjects and both the left and the right hemisphere, we calculated the slope of the percent signal change as a function of distance from the lesion border (see Table 1).

**Table 1:** **Mean percent signal change and its range (in brackets) for each perilesional “super-region” and the slope of the percent signal change as a function of distance from the lesion border**.

	Near “super-region”	Middle “super-region”	Far “super-region”	Slope
**Left Hemisphere**				
Patients	0.100 (−0.010–0.167)	0.127 (−0.068–0.267)	0.248 (−0.025–0.475)	0.074
Control subjects	0.064 (0.033–0.092)	0.023 (−0.062–0.150)	0.009 (−0.043–0.094)	−0.027
**Right Hemisphere**				
Patients	−0.035 (−0.216–0.194)	0.109 (0.001–0.207)	0.174 (−0.013–0.309)	0.104
Control subjects	0.085 (−0.001–0.155)	0.052 (0.071–0.126)	0.016 (−0.092–0.079)	−0.034

In the left hemisphere, the descriptive analyses demonstrate that patients tended to have a higher percent signal change than control subjects irrespective of the distance to the lesion border. Additionally, whereas the percent signal change tended to increase as a function of distance to lesion in patients, the percent signal change tended to decrease as a function of distance to lesion in the neurologically healthy subjects. In the right hemisphere, on the other hand, patients showed a lower percent signal change than control subjects in the near perilesional “super-region”, whereas they showed a higher percent signal change than control subjects in the middle and far perilesional “super-regions”. Additionally, whereas the percent signal change increased as a function of distance to lesion in the patients, the percent signal change tended to decrease as a function of distance to lesion in the control subjects.

In light of the large negative percent signal change close to the lesion border in patient 3, we used MarsBar to calculate the peristimulus time histograms to determine whether the timing of these negative values were comparable to the timing of the hemodynamic response function (HRF) peak obtained in the control subjects. These histograms show that the negative percent signal change values displayed by this patient occur around 8 s after the stimulus, which is similar to the timing of the peak in the peristimulus time histograms of the control subjects.

For a statistical analysis of these data, we likewise collated the signal over each four adjacent perilesional regions creating three perilesional “super-regions” for each patient in each hemisphere. Subsequently, we generated a mean score for the three control subjects assigned to each patient. Thus, for each patient and each perilesional “super-region” we obtained one percent signal change score for the patient and one averaged percent signal change score for the controls for both the right and the left hemisphere. With these percent signal change scores we performed a two (group: patient or controls) by three (distance: near, middle or far “super-region”) mixed design ANOVA in each hemisphere, using a Bonferroni correction for multiple comparisons.

In the left hemisphere, the ANOVA showed no main effect of either group (*F*_(1,4)_ = 1.403, *p* = 0.302) or distance (*F*_(2,8)_ = 1.00, *p* = 0.410), but a close to significant interaction between group and distance (*F*_(2,8)_ = 3.192, *p* = 0.096). Whereas the percent signal change tended to increase with increasing distance to the lesion in patients, the percent signal change tended to decrease with increasing distance to lesion in control subjects.

In the right hemisphere, the ANOVA demonstrated a significant interaction between group and distance (*F*_(2,8)_ = 12.283, *p* = 0.004). Follow-up one-way ANOVAs showed that the main effect of distance was close to significance in both patients (*F*_(2,4)_ = 7.649, *p* = 0.086) and controls (*F*_(2,4)_ = 10.169, *p* = 0.054). However, whereas in patients, the percent signal change tended to increase with increasing distance to the lesion, in control subjects, the percent signal change tended to decrease with increasing distance to the lesion.

The method we used to calculate the percent signal change assumes that the signal change evoked by the task events scales with the average signal of the region of interest (baseline signal). In other words, the evoked signal change is assumed to be proportional to the baseline signal. As a consequence, the signal change evoked by the task events is traditionally divided by the baseline signal to account for potential differences in the baseline signal between multiple regions of interest. While this is a generally accepted assumption and procedure, it is nevertheless possible that in our study the signal change evoked by the task events was not proportional to the baseline signal. Should this be the case, we would have overestimated the percent signal change in areas with a lower baseline and underestimated the percent signal change in areas with a higher baseline. To account for this possibility, we additionally calculated both the, non-proportional signal change evoked by the task events (i.e., the signal change undivided by the baseline signal) and the baseline signal in each region of interest. This revealed highly similar results regardless of whether the signal change was assumed to be proportional to the baseline signal (original analysis reported in the Materials and Methods Section) or not (additional analysis). Inspection of the baseline values of the regions of interest revealed that this was due to the fact that the baseline values were relatively constant in the different regions of interest.

## Discussion

Our findings suggest that acute stroke might systematically influence BOLD signals in structurally intact perilesional brain areas. When comparing the BOLD percentage signal change between patients with acute cortical damage and healthy elderly controls, we observed an abnormal interhemispheric balance consisting of lower levels of percent signal change in the damaged right hemisphere in two of our three patients. This abnormal interhemispheric balance was particularly pronounced near the lesion border and shows striking similarities to that reported by Corbetta et al. (2005) and—mirror-inverted for the opposite hemisphere—by Saur et al. (2006). However, although in our right hemisphere damaged patients there was a decrease in BOLD signal in the injured hemisphere similar to that reported by Corbetta et al. (2005), our participants did not exhibit spatial neglect (neither experimentally nor clinically), nor were they tested with a fMRI paradigm sensitive to spatial attention deficits, nor did they exhibit any deficits in the present (visual orientation judgment) task.

Both Corbetta et al. (2005) and Saur et al. (2006) concluded that the interhemispheric imbalance of BOLD signal observed in their patients underlies (part of) their behavioral defect. In the right hemisphere patients studied by Corbetta et al. (2005), a pathological interhemispheric “push-pull” pattern of attentional orienting was assumed to contribute to spatial neglect. In the left hemisphere patients studied by Saur et al. (2006), an upregulation of the right inferior frontal cortex was discussed to either represent real language processing and/or increased traffic in a relay station, possibly reflecting reduced trans-hemispheric inhibition. In theory, our results could likewise reflect a dysregulation of the function represented in the investigated brain regions. However, the behavior of our patients was not significantly different from the behavior of controls. This suggests that if a functional disruption did occur, it was mild enough not to have had any measurable effect on behavior. Moreover, our finding of an abnormal interhemispheric balance was specific to areas of the brain located close to the lesion and was thus expressed by different brain regions in the different patients (as the location of the lesion varied over the patients). Thus, while we cannot completely exclude—based on the small sample size—the possibility that our patients suffered from a discrete (and therefore here not measurable) behavioral impairment, the specificity of our effect to areas close to the lesion border, but variation of our effect in brain areas affected in the different patients, is difficult to explain in the context of a general behavioral deficit. Instead, we think our results reflect, at least in part, an abnormal neurovascular coupling after stroke due to impaired cerebrovascular reactivity.

An acute reduction of cerebral perfusion pressure during an ischemic stroke results in a decrease of cerebral blood flow (Dirnagl and Pulsinelli, 1990). When cerebral blood flow drops sufficiently, compensation mechanisms consisting of autoregulatory vasodilation and increased oxygen extraction are triggered to avoid irreversible brain damage (Derdeyn et al., 2002). This vasodilation results in impaired cerebrovascular reactivity since due to the pre-existing vasodilation, cerebral vessels are not able to dilate further in response to a vasodilatory stimulus. As the BOLD response fundamentally relies on an increase in regional blood flow after a transient increase in neuronal activity (Ogawa et al., 1990, 1992), BOLD responses have unsurprisingly been shown to be abnormal in stroke patients with impaired cerebrovascular reactivity (Carusone et al., 2002; Röther et al., 2002; Hamzei et al., 2003; Rossini et al., 2004; Krainik et al., 2005; Murata et al., 2006; Amemiya et al., 2012) despite the demonstrable presence of a transient increase in neuronal activity (Rossini et al., 2004). Moreover, impaired cerebrovascular reactivity has been shown to predict abnormal BOLD responses in patients both with (Hamzei et al., 2003; Amemiya et al., 2012) and without (Rossini et al., 2004; Krainik et al., 2005) arterial stenosis/occlusion.

Importantly, Richardson et al. (2011) found that cerebral perfusion was impaired close to the lesion border in their chronic stroke patients. Given the relationship between a decrease in cerebral blood flow and subsequent vasodilation-mediated impaired cerebrovascular reactivity (Derdeyn et al., 2002) and, correspondingly, observations of a co-occurrence of impaired cerebral blood flow and impaired cerebrovascular reactivity (Carusone et al., 2002; Murata et al., 2006; Amemiya et al., 2012), it seems reasonable to suggest that cerebrovascular reactivity might likewise be particularly impaired near the lesion border. Moreover, in situations where cerebral perfusion is only slightly impaired, this might result in impaired BOLD effects secondary to impaired cerebrovascular reactivity and neurovascular coupling in the presence of intact neuronal activity. Of course, when cerebral perfusion is sufficiently severely impaired, neuronal function will ultimately suffer, leading to a behavioral impairment (e.g., Karnath et al., 2005; Ticini et al., 2010). In theory, either misery perfusion or luxury perfusion could disrupt the normal cerebrovascular reactivity. One could consider misery perfusion where the reduced perfusion requires constant dilation even during functional rest. Likewise, tissue receiving luxury perfusion would not require any changes in blood flow even during active states. A possible mechanism for disrupted cerebrovascular reactivity and subsequent abnormal BOLD effects close to the lesion border could be the leaking out of vasodilatory substances from the infarct in the context of peri-infarct gliosis (Martin et al., 2001; D’Esposito et al., 2003; Barreto et al., 2011).

Both Corbetta et al. (2005) and Saur et al. (2006) report asymmetric interhemispheric effects in areas of the brain close to the lesion border. Our results suggest that precisely these areas in the lesioned hemisphere might display abnormal BOLD effects (and thus interhemispheric asymmetries) secondary to impaired cerebrovascular reactivity. Nevertheless, the timing of the BOLD fMRI measurements was somewhat different between studies: the measurements in our patients took place in the acute stage 2–6 days after the stroke occurred. Whereas this timing is comparable with the timing of the first measurements reported by Saur et al. (2006) that took place 0–4 days post stroke, Corbetta et al. (2005) assessed their patients for the first time approximately 3–4 weeks post stroke. Theoretically, it is possible that the abnormal interhemispheric balance that we and Saur et al. (2006) found within the first week post stroke, has resolved at 3–4 weeks post stroke. However, both impaired cerebrovascular reactivity (Rossini et al., 2004; Krainik et al., 2005) and impaired cerebral perfusion close to the lesion border (Richardson et al., 2011) have been reported in chronic patients, suggesting that abnormal BOLD effects secondary to impaired cerebrovascular reactivity persist into the chronic stroke stage.

An obvious limitation of our study is the small sample size. This means that our results are perhaps best seen as prelimimary observations. Moreover, the reduced BOLD effects were observed in areas of the brain close to the lesion border when comparing BOLD effects obtained in stroke patients to those obtained in neurologically healthy subjects. Thus, they are of relevance for the studies such as those by Corbetta et al. (2005) and by Saur et al. (2006) since the interhemispheric imbalance they reported both partly occurs in regions close to the lesion border and their homologues and was obtained by comparing stroke patients to neurologically healthy subjects. However, we wish to note that our present conclusions do not necessarily generalize to BOLD effects obtained far from the lesion border, in, e.g., a different cerebrovascular territory from the lesion, and/or in the other hemisphere. BOLD fMRI findings in stroke patients in such areas remote from the lesion might allow valid conclusions concerning neuronal function, provided the performance of patients who suffer from a particular deficit (e.g., aphasia, spatial neglect, etc.) is compared to the performance of stroke patients with similar injuries in the same cerebrovascular territory but who do not exhibit the behavioral deficit under study to control for general effects of a stroke not related to the symptom of interest. Valid conclusions might also be drawn from situations where the patient can serve as his/her own control in a within-subject comparison. A demonstration of a normal BOLD effect in a given region in a given patient in response to a certain condition strongly suggests intact neurovascular coupling in that region. A subsequent demonstration of a reduced BOLD effect in the same region of the same patient in a different condition can then not simply be attributed to abnormal neurovascular coupling in that region.

Our findings suggest neuroscientists need to exercise caution when interpreting BOLD fMRI data acquired in patients with stroke, particularly in areas of the brain located close to the lesion border, since abnormal BOLD responses could not only reflect functional disruption of these regions, but also a decoupling of the neurovascular response (without changes in neuronal functioning and/or in behavior), or a combination of these two effects. Moreover, our findings highlight a clear need for complimentary techniques that can help determine whether structurally intact regions with an abnormal BOLD response are functionally intact or disrupted. Possible complimentary techniques include EEG and MEG. Despite the promise of BOLD fMRI, careful work will be required to understand how the physiological changes that occur following stroke influence BOLD signals, particularly those close to the lesion border. Signal changes detected by this technique may not necessarily reflect changes in the neural function (e.g., Rossini et al., 2004), nor necessarily influence the individuals’ behavior.

## Conflict of interest statement

The authors declare that the research was conducted in the absence of any commercial or financial relationships that could be construed as a potential conflict of interest.

## Footnotes

^1^http://www.fil.ion.ucl.ac.uk/spm/

^2^http://www.mricro.com/mricron

^3^http://www.glahngroup.org/Members/anderson/flair-templates

^4^http://marsbar.sourceforge.net/
